# Distinct influenza surveillance networks and their agreement in recording regional influenza circulation: Experience from Southeast Michigan

**DOI:** 10.1111/irv.12944

**Published:** 2021-11-25

**Authors:** Peter M. DeJonge, Arnold S. Monto, Ryan E. Malosh, Joshua G. Petrie, Hannah E. Segaloff, Erin McSpadden, Caroline Cheng, Latifa Bazzi, Amy Callear, Emileigh Johnson, Rachel Truscon, Emily T. Martin

**Affiliations:** ^1^ Michigan Influenza Center, Department of Epidemiology University of Michigan School of Public Health Ann Arbor Michigan USA

**Keywords:** influenza, sentinel surveillance, surveillance, syndromic surveillance

## Abstract

**Introduction:**

In Southeast Michigan, active surveillance studies monitor influenza activity in hospitals, ambulatory clinics, and community households. Across five respiratory seasons, we assessed the contribution of data from each of the three networks towards improving our overall understanding of regional influenza circulation.

**Methods:**

All three networks used case definitions for acute respiratory illness (ARI) and molecularly tested for influenza from research‐collected respiratory specimens. Age‐ and network‐stratified epidemic curves were created for influenza A and B. We compared stratified epidemic curves visually and by centering at seasonal midpoints.

**Results:**

Across all seasons (from 2014/2015 through 2018/2019), epidemic curves from each of the three networks were comparable in terms of both timing and magnitude. Small discrepancies in epidemics recorded by each network support previous conclusions about broader characteristics of particular influenza seasons.

**Conclusion:**

Influenza surveillance systems based in hospital, ambulatory clinic, and community household settings appear to provide largely similar information regarding regional epidemic activity. Together, multiple levels of influenza surveillance provide a detailed view of regional influenza epidemics, but a single surveillance system—regardless of population subgroup monitored—appears to be sufficient in providing vital information regarding community influenza epidemics.

## 1 INTRODUCTION

Influenza surveillance is a vital component of effective public health practice during seasonal influenza epidemics, which can be associated with upwards of 35 million illnesses in the United States each year.^1^ During the “flu season,” surveillance data are used to concurrently assess epidemic trajectory, determine the most at‐risk populations, and provide interim guidance regarding intervention.^2^, ^3^, ^4^, ^5^ It is therefore indispensable that surveillance data are timely and valid reflections of the community's underlying influenza epidemic.

A wide range of surveillance systems is used to track seasonal influenza epidemics, based on everything from over‐the‐counter medication purchases, to school and workplace absenteeism, and even internet search results.^6^, ^7^, ^8^, ^9^, ^10^ The most traditional and representative data are generated by active surveillance for confirmed influenza cases, though there are many methods for this as well.^11^, ^12^ In Southeast Michigan, three prospective surveillance systems exist to actively capture influenza cases from regional households, ambulatory clinics, and tertiary care hospitals. While the primary purpose of each network is to assess seasonal vaccine effectiveness in these distinct populations, their data are also used to monitor epidemic trends in the region. A better understanding of how these systems agree or disagree epidemiologically has important implications regarding how the choice of surveillance population influences an overall interpretation of the regional influenza epidemic.

In this investigation, we consolidated surveillance data from each of these distinct networks across five different influenza seasons. For one, we were interested in whether epidemic curves of any of the three networks displayed unique temporal patterns or features (e.g., characteristic timing of an epidemic peak or initial detection of epidemic spread). Second, we assessed whether there were any consistent patterns or relationships between the three networks (e.g., activity in one network foreshadowing activity in the other two). Finally, we considered how seasonal patterns in network activity aligned with knowledge of past influenza season characteristics.

## 2 METHODS

The Michigan Influenza Center at the University of Michigan operates three large studies which conduct prospective, active surveillance for influenza cases from community households, ambulatory clinics, and hospitals in Southeast Michigan (Table 1). Each network is designed to estimate vaccine effectiveness against a specific influenza outcome (i.e., community‐acquired, medically attended, and hospitalized disease). As a result, data captured by each of the three networks reflect the seasonal patterns of regional influenza circulation within three distinct source populations—each of which with unique characteristics like age, underlying health status, and experienced illness severity.

**TABLE 1:** Description of three prospective influenza surveillance studies in Southeast Michigan

	Hospital	Ambulatory	Household
Study Name	HAIVEN *(Hospitalized Adult Influenza Vaccine Effectiveness Network)*	MFIVE *(Michigan ‐ Henry Ford Influenza Vaccine Effectiveness study)*	HIVE *(Household Influenza Vaccine Evaluation study)*
Age eligibility	Participants ≥18 years old	Participants ≥6 months old	Households who use the University of Michigan health system with at least three members and one child <10 years old at enrollment
Illness eligibility	Patients who were recently hospitalized (≤72 h) for ARI ≤10 days' duration, broadly defined by admission diagnosis, with new onset cough	Clinical presentation with a recent ARI, defined as the presence of new cough ≤7 days' duration	Participants are instructed to report illness cases from anyone in the household, defined as the presence of two or more symptoms of ARI
Laboratory methods	Singleplex RT‐PCR	Singleplex RT‐PCR	Singleplex RT‐PCR
Geographic restrictions for this paper	Only patients seen at the main Michigan Medicine hospital	Only patients seen at Michigan Medicine affiliated clinics	Only community households that receive care from Michigan Medicine

Period of surveillance each season			
2014–2015	17 weeks (Nov 5 to Mar 6)	16 weeks (Nov 10 to Mar 5)	Year‐round, starting Oct 1
2015–2016	20 weeks (Nov 23 to Apr 15)	14 weeks (Jan 4 to Apr 14)	Year‐round
2016–2017	27 weeks (Oct 16 to Apr 28)	14 weeks (Jan 3 to Apr 14)	Year‐round
2017–2018	29 weeks (Oct 6 to Apr 28)	19 weeks (Nov 13 to Mar 30)	Year‐round
2018–2019	31 weeks (Oct 19 to May 24)	20 weeks (Dec 10 to May 3)	Year‐round

Enrollment sites assessed in study (or households/participants enrolled for HIVE)			
2014–2015	1 hospital	7 clinics	343 households/1435 individuals
2015–2016	1 hospital	5 clinics	226 households/992 individuals
2016–2017	1 hospital	5 clinics	297 households/890 individuals
2017–2018	1 hospital	5 clinics	291 households/1187 individuals
2018–2019	1 hospital	7 clinics	351 households/1115 individuals

This work utilizes data from five respiratory seasons, during which all networks were active and operational in their current form, starting during the 2014/2015 season and ending in 2018/2019. Enrollment and sampling procedures for all three studies were approved by the University of Michigan Medical School IRB.

### 2.1 Household

**FIGURE 1:**
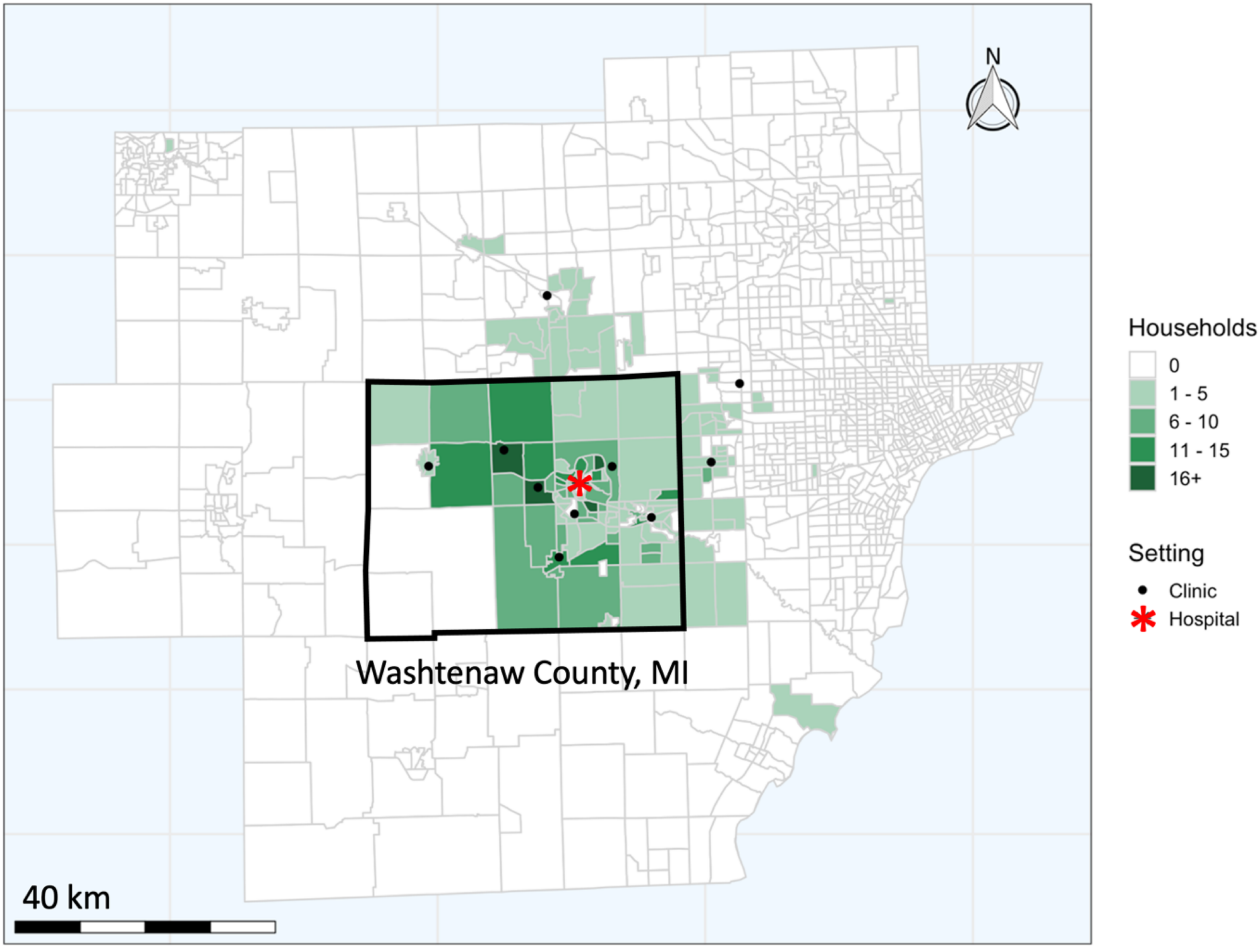
Location of households, ambulatory clinics, and the hospital system enrolled or previously enrolled in three prospective influenza surveillance studies across Southeast Michigan census tracts, 2014/2015 through 2018/2019

The *Household Influenza Vaccine Evaluation* study (HIVE) is an ongoing, prospective cohort study of community households, the majority of which are in Washtenaw County in Southeast Michigan (Figure 1).^13^, ^14^, ^15^, ^16^ While specific eligibility criteria changed over the course of the study, currently eligible households receive care from the Michigan Medicine healthcare system and have at least three members with at least one child <10 years old.^16^ Participants are queried weekly about the occurrence of acute respiratory illness (ARI) in any household member. An ARI episode is defined as the presence of two or more age‐specific symptoms of ARI, as described previously.^16^ Following an illness, participants visit the study clinic where combined throat and nasal swabs (nasal swabs only for children <3 years) are collected by study staff.

### 2.2 Ambulatory

As part of the US Flu Vaccine Effectiveness Network, the *Michigan‐Ford Influenza Vaccine Effectiveness* (MFIVE) study contributes data to annual estimates of influenza vaccine effectiveness in the ambulatory care setting.^17^, ^18^ Cases in ambulatory patients are identified from outpatient intake forms noting ARI symptoms and cough, with illness onset ≤7 days ago, and from individuals ≥6 months of age. If patients (or their legal guardians) agree to participate in the study, combined throat and nasal swabs (nasal swabs only for children <3 years) are collected by study staff; participants (or their legal guardians) are asked to complete a demographic questionnaire. During study years considered in this analysis, a majority of clinics were located in Washtenaw County, while three clinics were located in neighboring Livingston and Wayne Counties (Figure 1).

### 2.3 Hospital

The *Hospitalized Adult Influenza Vaccine Effectiveness Network* (HAIVEN) monitors vaccine effectiveness against influenza‐associated hospitalization in adults.^19^, ^20^, ^21^ In this study, staff use hospital intake logs and admission notes to find eligible participants, defined as: adult inpatients (≥18 years old) who recently presented to the hospital (≤72 h) with ARI symptoms, cough, and a reported illness onset ≤10 days ago. Similar to the ambulatory study, after enrollment and consent procedures, study staff collect a combined throat and nasal respiratory sample and complete a demographic questionnaire with the participant. Study recruitment occurs at the University of Michigan Hospital in Ann Arbor, Michigan (Figure 1).

### 2.4 Laboratory methods

Respiratory illness swabs collected from participants in all three surveillance networks were tested for influenza using reverse transcriptase polymerase chain reaction (RT‐PCR). The Influenza Division of the US Centers for Disease Control and Prevention (CDC) provided all primers, probes, and lab protocol for each study; these were designed for detection of universal influenza A and B, as well as their respective subtypes and lineages. All tests were performed at the Michigan Influenza Center laboratory.

### 2.5 Seasonality

We included all influenza positive cases that had been prospectively collected in each of the three networks from the 2014/2015 through 2018/2019 season. Notably, while the household study has operated year‐round starting October 1 of 2014, the ambulatory and hospital studies commence active surveillance at confirmation of regional influenza circulation each season, continue for at least 12 weeks, and may potentially end surveillance before complete termination of the epidemic in the community.

### 2.6 Epidemic comparisons

We created influenza epidemic curves for each network in each season. Curves were proportionally standardized as influenza cases reported each week divided by the network's total cases recorded that season; these curves were created separately for influenza A (aggregated H1N1, H3N2, and undetermined subtype) and influenza B (aggregated Yamagata, Victoria, and undetermined lineage). Cases of influenza A and B codetection contributed to both influenzas A and B epidemic curves.

One method of comparison in this analysis was based on the time period each season during which a network recorded the middle 50% of its respective influenza cases that season (i.e., 25% to 75% of all influenza A and B cases). We also compared curves by centering each network's total influenza epidemic curve to the seasonal midpoint of the overall region. These seasonal epidemic midpoints were defined as the week at which 50% of all influenza cases that season had been detected across all three networks. This adjustment was used to compare network timing in the context of the overall community. Finally, we stratified epidemic curves by age of participant at illness onset. Ages were categorized into four groups—0 to 6 years, 7 to 18 years, 19 to 54 years, and 55 years plus.

## 3 RESULTS

### 3.1 Case counts

Altogether, 13,028 ARI cases were recorded across the three networks from 2014/2015 through 2018/2019 in Southeast Michigan (Table 2). Among these, 2,371 (18.2%) samples were RT‐PCR‐positive for either influenza A or B. Influenza A and influenza B codetection was recorded in 27 cases over this time period (1.1%)—25 in the ambulatory network and 2 in the hospital network. Across all seasons, samples were positive for influenza in 1,434 of 4,989 (28.7%) ambulatory cases, 393 of 2,276 (17.2%) hospitalized cases, and 544 of 5,763 (9.4%) household cases. This ranked order of percent positivity was consistent across seasons—the highest percent positivity was always recorded in the ambulatory setting, followed by the hospital, and then the household network (Table 2).

**TABLE 2:** Case counts of influenza and acute respiratory illness (ARI) in each surveillance network across seasons

Network	Season	A	B	A(H1N1)	A(H3N2)	B (Victoria)	B (Yamagata)	Total influenza	Seasonal ARI	Seasonal ARI ‐ influenza positive	Overall ARI ‐ influenza positive
Ambulatory (*MFIVE*)	2014–15	254	12	1	251	1	12	266	1057	25.2%	28.7%
	2015–16	134	40	123	10	18	20	174	738	23.6%	
	2016–17	158	87	1	151	24	59	245	833	29.4%	
	2017–18	260	85	31	208	2	81	345	975	35.4%	
	2018–19	373	31	166	198	16	11	404	1386	29.1%	
Hospital (*HAIVEN*)	2014–15	119	11	0	114	0	11	130	754	17.2%	17.3%
	2015–16	47	3	0	4	1	1	50	313	16.0%	
	2016–17	47	21	0	45	1	20	68	462	14.7%	
	2017–18	71	23	4	57	0	21	94	371	25.3%	
	2018–19	51	0	19	28	0	0	51	376	13.6%	
Household (*HIVE*)	2014–15	166	46	0	166	11	34	212	1558	13.6%	9.4%
	2015–16	33	10	27	1	5	5	43	851	5.1%	
	2016–17	54	33	2	50	4	27	87	878	9.9%	
	2017–18	83	28	1	66	4	22	111	946	11.7%	
	2018–19	86	5	51	27	5	0	91	1530	5.9%	
All		1936	435	426	1376	92	324	2371	13,028	*N/A*	18.2%

### 3.2 Circulating virus

**FIGURE 2:**
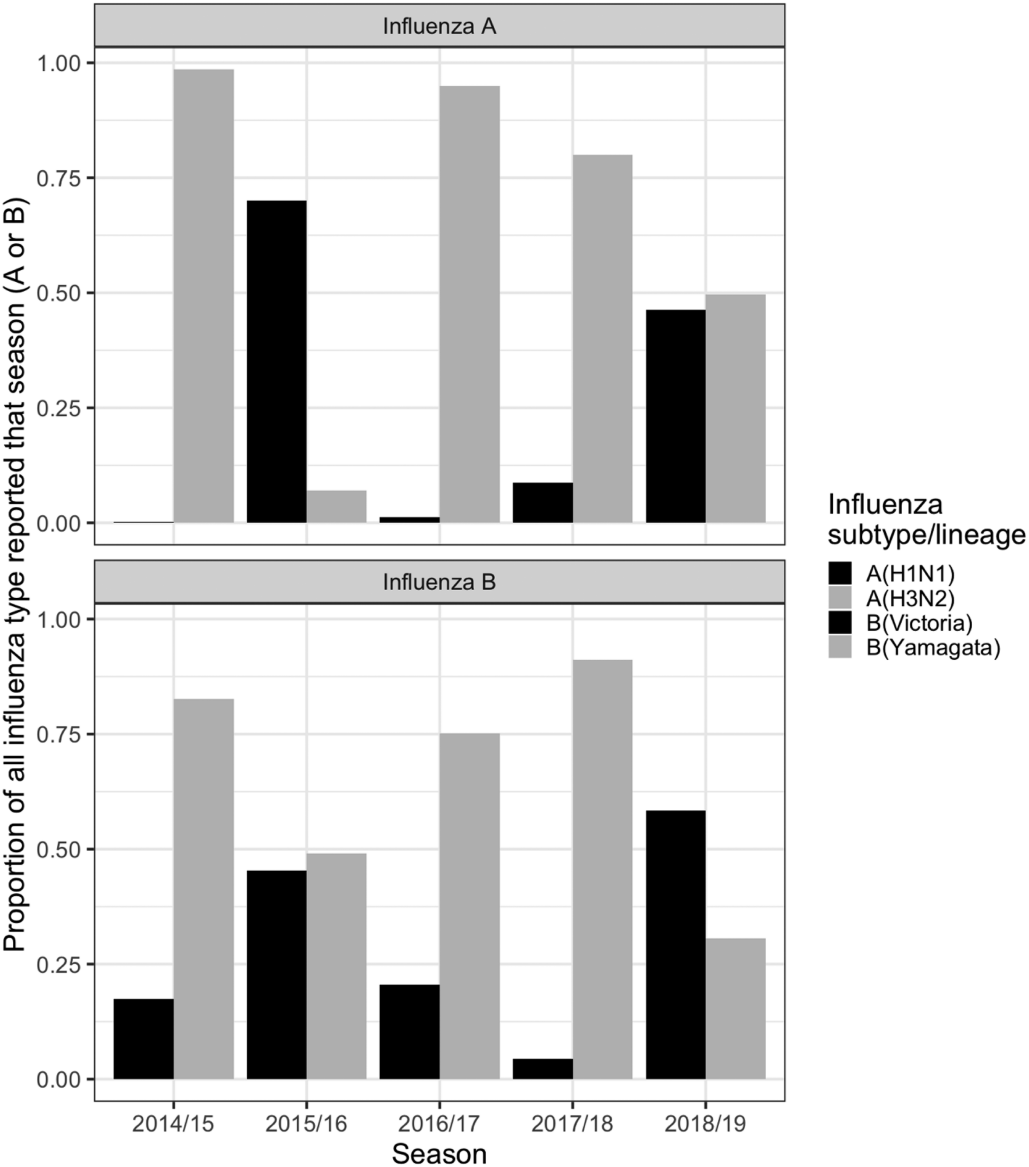
Detection of influenza A and B and their respective subtypes and lineages as a proportion of all cases, across five surveillance seasons

In all but 2018/2019, respiratory seasons were characterized by a single dominant influenza A subtype and B lineage. Figure 2 displays this phenomenon using aggregated data from all three networks and this pattern remained consistent when we stratified by network (data not shown). Three of five seasons were characterized by a dominant A(H3N2) virus (2014/2015, 2016/2017, and 2017/2018), while in the 2015/2016 season, A(H1N1) predominated. The 2018/2019 season displayed roughly equal proportions of A(H3N2) and A(H1N1) detections. For influenza B, B (Yamagata) was the dominant lineage in three surveillance seasons (2014/2015, 2016/2017, and 2017/2018); there was a considerable mix of both influenza B lineages in 2015/2016 and 2018/2019.

### 3.3 Seasonality

**FIGURE 3:**
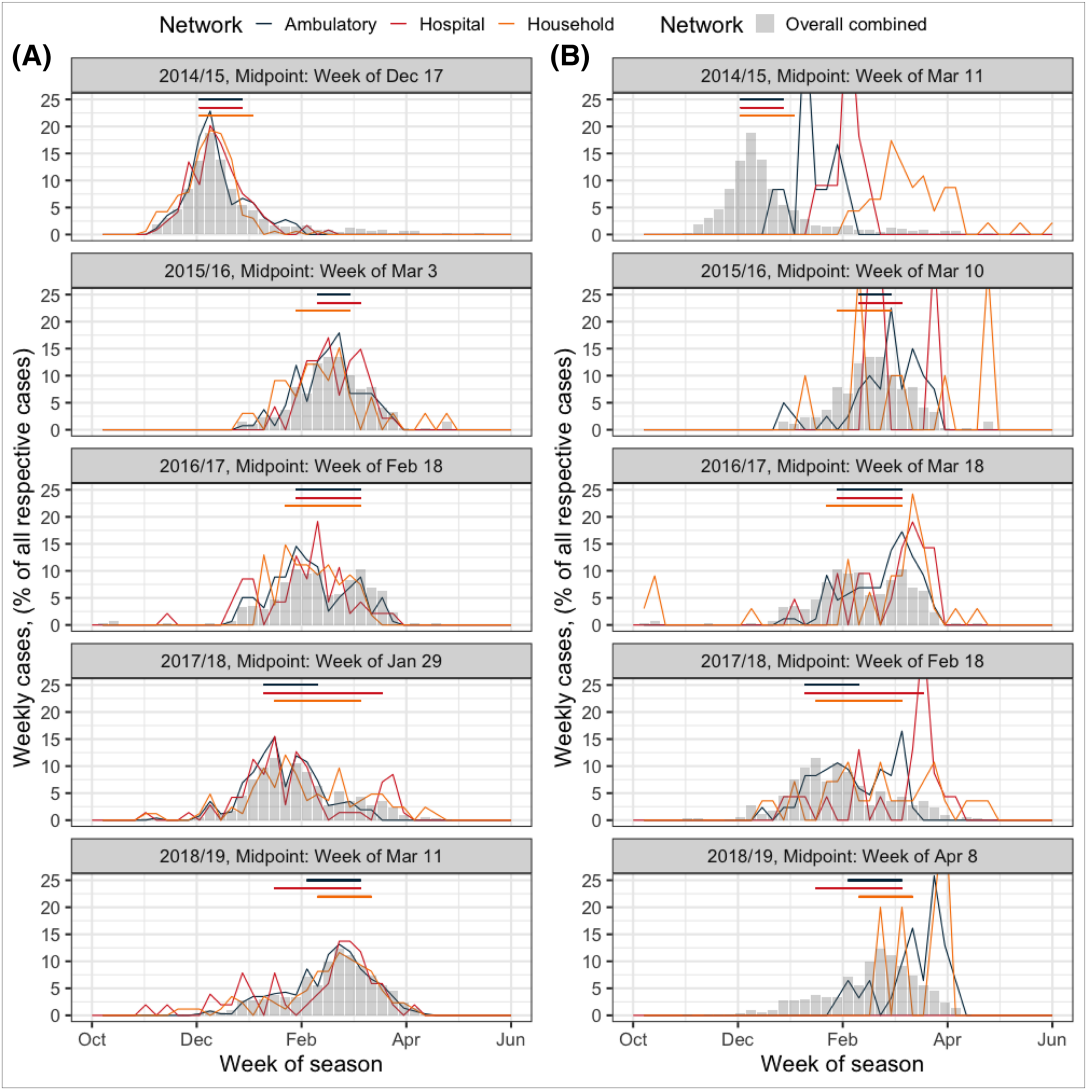
Overall epidemic curves for influenza A and B recorded by three surveillance networks of Southeast Michigan across five surveillance seasons. The left column (A) represents influenza A cases (aggregate of H1N1, H3N2, undetermined subtypes) and the right column (B) represents influenza B cases (aggregate of Victoria, Yamagata, undetermined lineages). The grey bars reflect the epidemic curve of all influenza cases from all networks reported that season. The colored lines reflect influenza A and B epidemics of each of the three networks. Y‐axis units represent the network‐standardized weekly number of cases reported, as a proportion of all cases reported in the network that season. Horizontal, colored lines are equivalent within rows and represent the period during which the middle 50% of all influenza cases (influenza A and B combined) were reported to a given network that season

Most ARI in the region generally occurred between January and April (Figure S1). Seasonality of influenza was pronounced and captured similarly from year‐to‐year by each of the three networks (Figure 3). The influenza epidemic in all five seasons began with influenza A circulation, which tended to reach its midpoint in late February. Influenza B activity followed and generally reached its midpoint 1 to 4 weeks after the influenza A midpoint. The exception to this was in 2014/2015, when influenza A activity was early and prolonged in all three networks, reaching its midpoint in mid‐December—much earlier than in other seasons. We saw no evidence of any consistent sequence or pattern in the order of network activity; for example, no one network was persistently peaking prior to the others.

In each season, the middle 50% of each network's total influenza cases was detected over roughly the same interval, which generally occurred 6 weeks after initial epidemic circulation and lasted for around 6 weeks (represented by the colored horizontal lines in Figure 3). In all but the notably early season of 2014/2015, the bulk of this 6‐week period happened during the month of February. In two seasons, 2017/2018 and 2018/2019, the hospital's middle 50% of all influenza cases lasted markedly longer than that of either the household or ambulatory networks.

**FIGURE 4:**
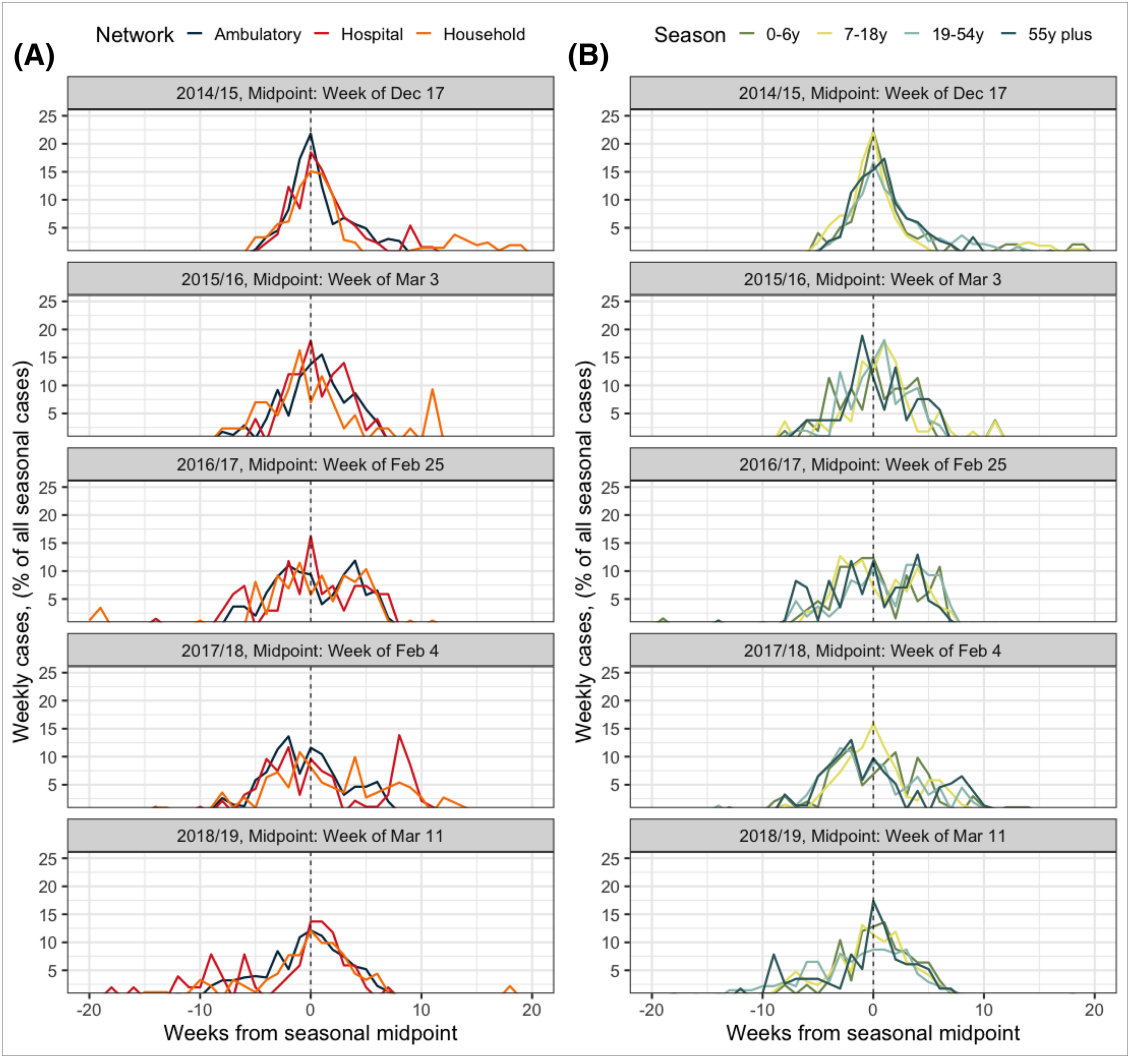
Epidemic curves centered at overall community midpoint in Southeast Michigan, stratified by surveillance network and age group across five surveillance seasons. The left column (A) represents epidemic curves of all influenza A and B, stratified by network. The right column (B) represents epidemic curves of all influenza A and B, stratified by age group of individual. Y‐axis units represent the network (or age group) standardized weekly number of cases reported, as a proportion of all cases reported in the network (or age group) that season. The seasonal midpoint is defined as the calendar week at which 50% of a season's total influenza cases (A and B, across all three networks) had been reported. The week is noted in the facet title for each row

Epidemic activity recorded by all three networks appeared broadly similar when evaluating curves centered at the community's epidemic midpoint for overall influenza each season (i.e., the week at which the combined total of household, ambulatory, and hospital cases reached 50% of all seasonal cases, Figure 4). In general, the beginning of each network's influenza activity was recorded about eight to 10 weeks in advance of the community midpoint. The exception to this was the 2014/2015 season, when all three networks displayed nearly identical rates of sharp growth about 5 weeks in advance of the community midpoint (Figure 4A). The 2014/2015 season also displayed the longest epidemic decline, particularly in the household study, with influenza persistence noted for nearly 5 months after the calculated community midpoint. Regional epidemic patterns were also similar when stratified by age group across seasons (Figure 4B).

## 4 DISCUSSION

Our study compared epidemic influenza data from three distinct surveillance networks operating within a single geographic region in the US and found that there was considerable epidemiologic agreement between the three networks across seasons. Analogues of HIVE, HAIVEN, and MFIVE exist in other parts of the United States, but it is unusual to have three active surveillance platforms operating simultaneously within the same area. This is the first time that epidemic data from these three networks has been directly compared.

Prior to this work, we suspected that influenza epidemic curves detected by each network would have distinguishing characteristics because of the unique, underlying surveillance populations. For instance, because more mild illnesses are detected in the HIVE household study compared with the HAIVEN hospital network, we anticipated differences in the timing and magnitude of HIVE and HAIVEN influenza epidemics. Instead, we found that influenza epidemics in Southeast Michigan were recorded similarly by each of the three networks across all five seasons. All three networks (i) captured comparable distributions of circulating influenza A subtypes and B lineages, (ii) reported both the peak and bulk of influenza activity at approximately the same week each season, and (iii) displayed a similar epidemic progression, represented by the shape of their epidemic curves.

Each of the three networks uses influenza surveillance as a means to an end; the primary purpose of each study is to gauge influenza vaccine effectiveness against different outcomes (community‐acquired, medically‐attended, and hospitalized disease).^16^, ^20^, ^22^ Surveillance data from the household network has also been used to provide estimates and predictors of vaccine uptake, as well as influenza transmission parameters.^15^, ^23^ Therefore each individual network is necessary for a better understanding of influenza prevention in these three distinct settings.

A closer look at our results, specifically at the few instances when the three curves did not agree, also attests to the unique information provided by each of the three surveillance systems. When data from the three systems are considered all together, we are provided with a more holistic view of regional influenza epidemics. Below, we highlight three seasons in which discrepancies in network epidemics helped to retrospectively explain characteristics of seasonal influenza, including vaccine effectiveness, disease severity, and circulating strains.

First, the 2014/2015 season is notable in our results for the early activity and rapid escalation to peak of all three networks. This matches national summaries of that season.^24^ Our observed peak in Southeast Michigan occurred about a week earlier than the reported national peak (week of December 27). The rapid escalation nationwide is likely due to the fact that the dominant A(H3N2) virus was antigenically distinct from its counterpart in the vaccine that year. Of all seasons assessed in our study, this season was associated with the lowest adjusted overall vaccine effectiveness. The CDC's final estimated vaccine effectiveness against influenza‐associated ARI was 19% for all age groups and only 1% against the circulating A(H3N2) strain.^24^, ^25^ The considerable mismatch in vaccine strain and circulating strain may have been an important factor contributing to the rapid progression of the epidemic in Southeast Michigan, much like the rest of the United States. We point out that around 70% of the HIVE household study population reported vaccination in 2014/2015, which was much higher than the 49.2% of the US population older than 6 months of age who were vaccinated.^26^ Our results underscore the fact that influenza can still transmit rapidly in a population with high uptake of a poorly matched vaccine.

The 2014/2015 season also attests to the importance of extending surveillance periods outside of pre‐specified timeframes—a limitation of both the hospital and ambulatory networks. In 2014/2015, the household network was the only system to capture the lingering influenza B transmission; this epidemic persistence was a notable feature of the CDC's seasonal summary that year.^24^ Because surveillance is stopped at the designated end of the influenza epidemic for both ambulatory and hospital networks (determined during each season in collaboration with the CDC), they failed to capture the community outbreak of influenza B which lasted through early June. For complete surveillance of the season's entire influenza epidemic, a less stringent “end‐of‐season” definition or an extended surveillance period would have been necessary.

Second, we point to the 2017/2018 season, where influenza transmission was detected for a prolonged period of time in the hospital system and even accelerated to a secondary peak in late March (Figure 3). Compared with other seasons, the hospital system's epidemic in 2017/2018 was more distinct than that of the household or ambulatory system. In particular, the middle 50% of all hospital cases was recorded over a period of 11 weeks (compared with the 5 weeks and 8 weeks of our household and ambulatory systems, respectively). Alongside this fact, 25% of all eligible ARI admissions in the hospital that season tested positive for influenza—considerably higher than any other season's percent positive value, which ranged from 14% to 17% (Table 2). Both of these observations reflect the severity of the influenza season that year. Despite a well‐matched vaccine with an effectiveness of 39% against all influenza, this season was nationally notable for its dramatic illness severity.^27^ Based on the CDC's standard method of categorizing the severity of seasonal influenza epidemics from 2003/2004 onward, the 2017/2018 season was the first season to classify as high severity across all age groups; influenza‐associated hospitalization rates were the highest ever recorded through the national FluSurv‐NET system.^28^

Finally, the 2018/2019 season was notable for its recorded persistence of influenza circulation. Generally, in most seasons that we assessed, cases recorded in all three networks began to rise around 10 weeks in advance of the seasonal midpoint and then declined over a period of months. This influenza epidemic progression agrees with previous work.^4^ The 2018/2019 season does not follow this pattern, however. Instead, all three networks slowly grew to their peaks that year over the course of 3 months; CDC's annual summary corroborates this finding, which reported that the 2018/2019 season was the longest epidemic of the past 10 years.^29^ Nationally, outpatient visits for influenza‐like illness were recorded above the national baseline for 21 consecutive weeks. As was observed in our Southeast Michigan networks, this observation may have been due to a dual influenza A season, where a surge of influenza A(H1N1) in the early winter was followed by a subsequent wave of influenza A(H3N2). In this way, A(H3N2) replaced a waning A(H1N1) season and maintained steady influenza transmission in a still‐susceptible population (Figure S2). Figure 1 reflects this dual‐A season regionally, where 2018/2019 was the only season with a non‐dominant influenza A strain detected.

A reliable early warning sign of impending influenza epidemics would be indispensable for public health preparedness and resource allocation—particularly in the event of a novel pandemic strain.^30^ Because research has established school as a driver of communicable disease spread and children as important introducers of virus to their households, we initially thought that a season's influenza activity would be first picked up by our household study.^31^, ^32^, ^33^, ^34^, ^35^, ^36^, ^37^ This was not the case. While households reported ARI throughout the year (Figure S1), there was no persistent early reporting of confirmed influenza in our household network (or the other two networks, for that matter). We were also interested in whether a particular age group would experience influenza activity before all others; in one past study, children experienced higher influenza risk before the community epidemic midpoint compared to older age groups.^38^ After we stratified epidemic curves into four age groups, we found no evidence that influenza activity was occurring persistently earlier in children or any other age group (Figure 4B). This is not to say that children are not important purveyors of influenza transmission in a community. Instead, our regionally‐focused data suggest that children did not serve as the proverbial “canaries in the coal mine” for community outbreaks during those five seasons.^39^

While considering data from all three systems together provided us with a more comprehensive view of the overall, regional epidemic our results show that epidemics detected by the three networks are largely comparable. Epidemic peaks, troughs, viruses detected, and epidemic duration appeared largely similar across all three networks in each season, regardless of seasonally circulating influenza types. This general agreement between networks should be considered a point of reassurance for local public health departments that may not have access to data from three distinct, prospective influenza surveillance networks. For real‐time responsiveness, our results show that household‐, clinic‐, or hospital‐based influenza surveillance in a community can provide local public health experts with reliable information and could be used to guide meaningful response and preparedness efforts in a timely fashion.

## AUTHOR CONTRIBUTIONS

**Peter M. DeJonge:** Conceptualization; data curation; formal analysis; methodology; software; visualization. **Arnold S. Monto:** Conceptualization; funding acquisition; project administration; supervision. **Ryan E. Malosh:** Data curation; methodology; resources. **Joshua G. Petrie:** Data curation; methodology; resources; supervision. **Hannah E. Segaloff:** Data curation; methodology. **Erin McSpadden:** Data curation; project administration; resources. **Caroline Cheng:** Formal analysis; resources; software. **Latifa Bazzi:** Data curation; project administration; software. **Amy Callear:** Data curation; project administration; software. **Emileigh Johnson:** Data curation; project administration; resources. **Rachel Truscon:** Data curation; investigation; resources. **Emily T. Martin:** Conceptualization; data curation; formal analysis; funding acquisition; investigation; methodology; project administration; resources; supervision.

### PEER REVIEW

The peer review history for this article is available at https://publons.com/publon/10.1111/irv.12944.

## Supporting information


**Figure S1:** Seasonal reports of acute respiratory illness (ARI) across three surveillance networks in southeast Michigan
Lines represent weekly sums of ARI reported to each network

**Figure S2:** Epidemic curves for influenza A subtypes during the 2018/19 season
The colored lines reflect influenza A and B epidemics of each of the three networks. Y‐axis units represent the network‐standardized weekly number of cases reported, as a proportion of all cases reported in the network that season.

## Data Availability

The data that support the findings of this study are available from the corresponding author upon reasonable request.
