# Supplementary material for: Distinct influenza surveillance networks and their agreement in recording regional influenza circulation: Experience from Southeast Michigan
Source: Influenza Other Respir Viruses. 2021 Nov 25;16(3):521–31. doi: 10.1111/irv.12944 (PMC8983886; doi:10.1111/irv.12944)
Supplement: Supplementary file 1 — Figure S1: Seasonal reports of acute respiratory illness (ARI) across three surveillance networks in southeast Michigan Lines represent weekly sums of ARI reported to each network [file IRV-16-521-s002.docx]

### Supplementary Figure 1: Seasonal reports of acute respiratory illness (ARI) across three surveillance networks in southeast Michigan

**
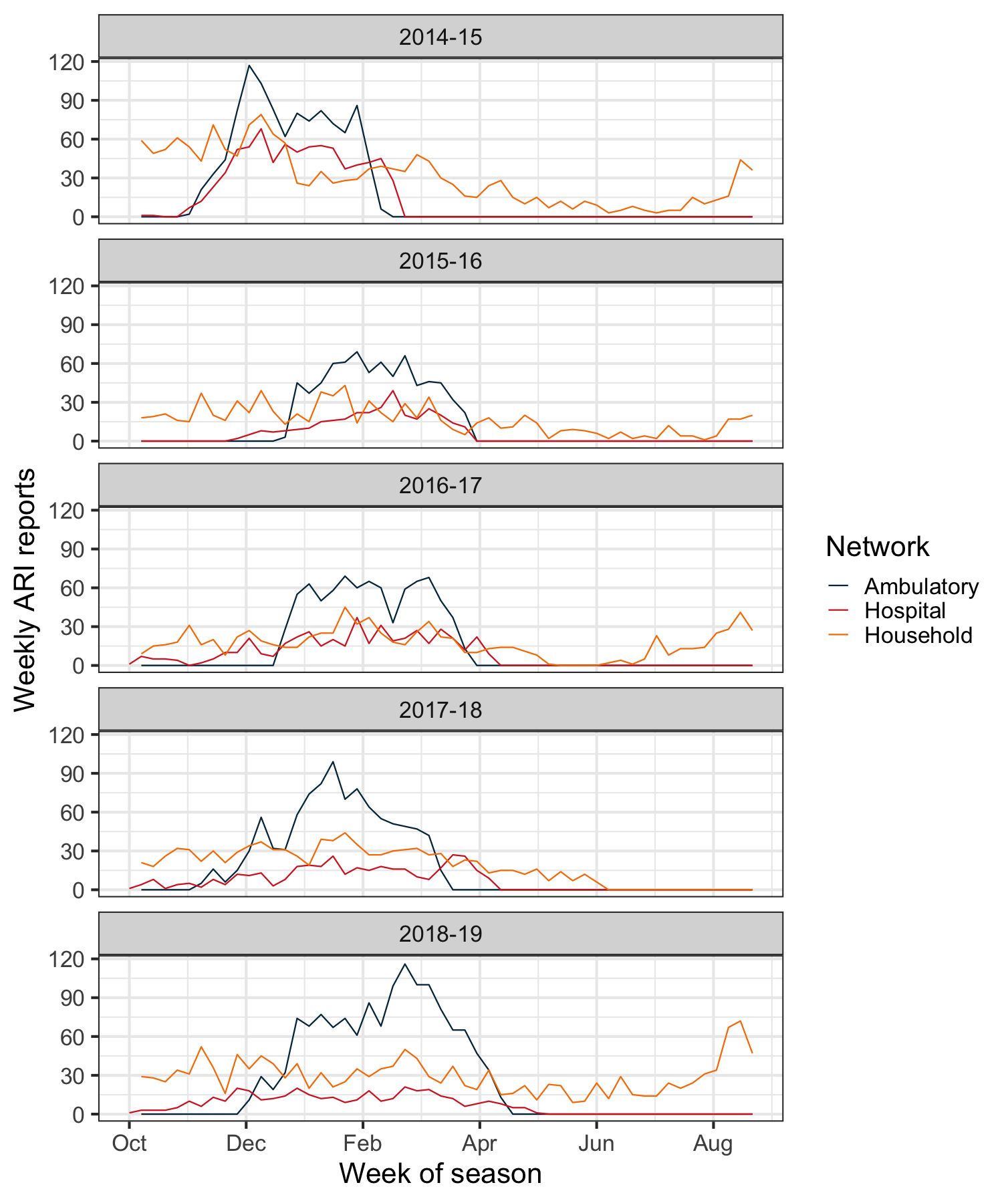
**

Lines represent weekly sums of ARI reported to each network
