# Supplementary material for: Distinct influenza surveillance networks and their agreement in recording regional influenza circulation: Experience from Southeast Michigan
Source: Influenza Other Respir Viruses. 2021 Nov 25;16(3):521–31. doi: 10.1111/irv.12944 (PMC8983886; doi:10.1111/irv.12944)
Supplement: Supplementary file 2 — Figure S2: Epidemic curves for influenza A subtypes during the 2018/19 season The colored lines reflect influenza A and B epidemics of each of the three networks. Y‐axis units represent the network‐standardized weekly number of cases reported, as a proportion of all cases reported in the network that season. [file IRV-16-521-s001.docx]

### Supplementary Figure 2: Epidemic curves for influenza A subtypes during the 2018/19 season


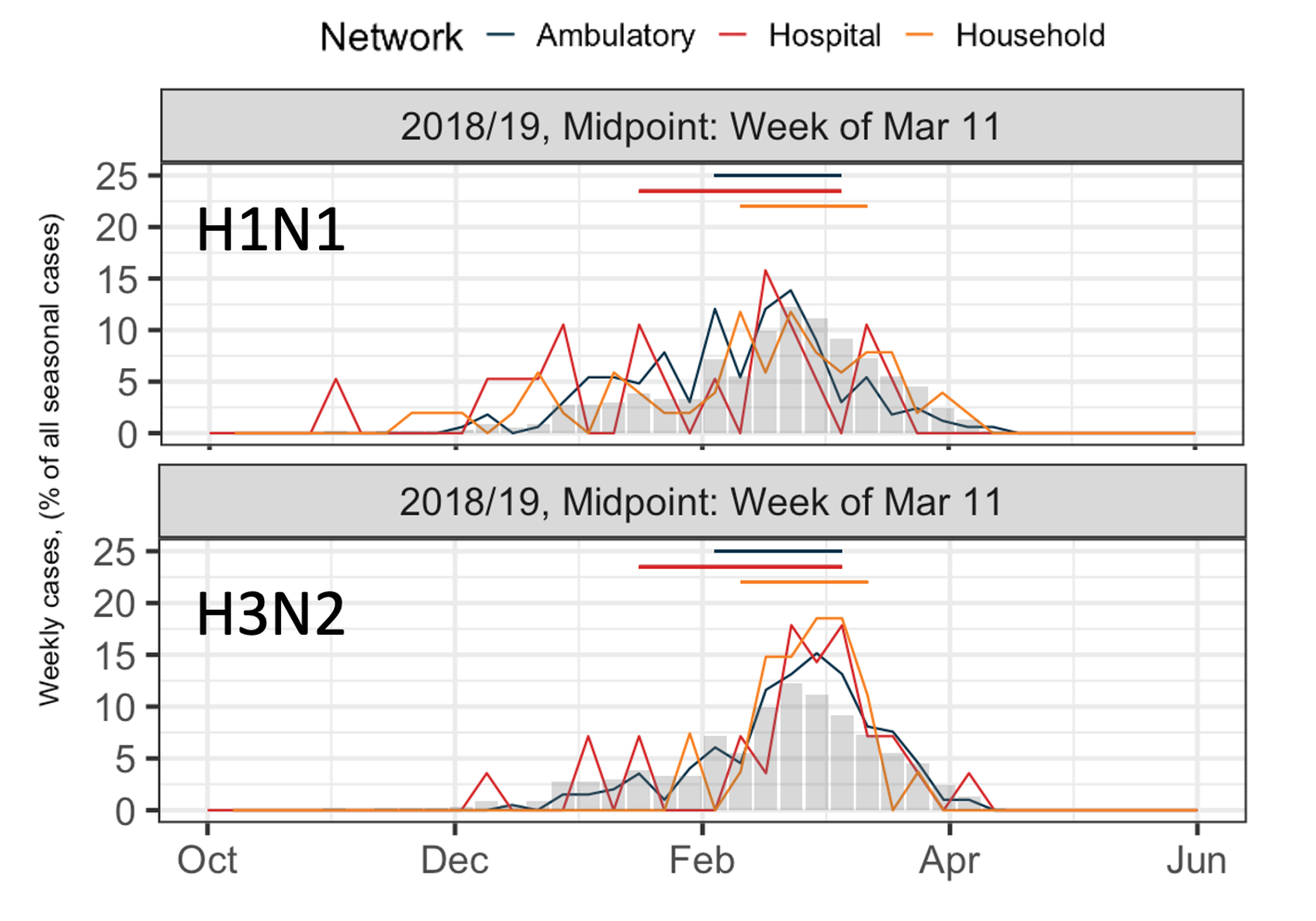


The colored lines reflect influenza A and B epidemics of each of the three networks. Y-axis units represent the network-standardized weekly number of cases reported, as a proportion of all cases reported in the network that season.
